# Metabolic stress induces a Wnt-dependent cancer stem cell-like state transition

**DOI:** 10.1038/cddis.2015.171

**Published:** 2015-07-02

**Authors:** E Lee, J Yang, M Ku, N H Kim, Y Park, C B Park, J-S Suh, E S Park, J I Yook, G B Mills, Y-M Huh, J-H Cheong

**Affiliations:** 1 Department of Radiology, Yonsei University College of Medicine, Seoul 120-752, Republic of Korea; 2 Nanomedical National Core Research Center, Yonsei University, Seoul 120-749, Republic of Korea; 3 Yonsei-KRIBB Medical Convergence Research Institute, Yonsei University Health System, Seoul, Korea; 4 Brain Korea 21 PLUS Project for Medical Science, Yonsei University, Seoul 120-752, Republic of Korea; 5 Department of Oral pathology, Oral Cancer Research Institute, Yonsei University College of Dentistry, Seoul 120-752, Republic of Korea; 6 Severance Biomedical Science Institute (SBSI), Seoul 120-752, Republic of Korea; 7 Department of Systems Biology, MD Anderson Cancer Center, Houston, TX, USA; 8 Department of Surgery, Yonsei University College of Medicine, Seoul 120-752, Republic of Korea; 9 Department of Biochemistry & Molecular Biology, Yonsei University College of Medicine, Seoul 120-752, Republic of Korea

## Abstract

Reciprocal interactions between cancer cells and the tumor microenvironment drive multiple clinically significant behaviors including dormancy, invasion, and metastasis as well as therapy resistance. These microenvironment-dependent phenotypes share typical characteristics with cancer stem cells (CSC). However, it is poorly understood how metabolic stress in the confined tumor microenvironment contributes to the emergence and maintenance of CSC-like phenotypes. Here, we demonstrate that chronic metabolic stress (CMS) in a long-term nutrient deprivation induces a Wnt-dependent phenoconversion of non-stem cancer cells toward stem-like state and this is reflected in the transcriptome analysis. Addition of Wnt3a as well as transfection of dominant-negative Tcf4 establishes an obligatory role for the Wnt pathway in the acquisition of CSC-like characteristics in response to metabolic stress. Furthermore, systematic characterization for multiple single cell-derived clones and negative enrichment of CD44+/ESA+ stem-like cancer cells, all of which recapitulate stem-like cancer characteristics, suggest stochastic adaptation rather than selection of pre-existing subclones. Finally, CMS in the tumor microenvironment can drive a CSC-like phenoconversion of non-stem cancer cells through stochastic state transition dependent on the Wnt pathway. These findings contribute to an understanding of the metabolic stress-driven dynamic transition of non-stem cancer cells to a stem-like state in the tumor metabolic microenvironment.

Studies of neoplastic tissues have provided evidence for self-renewing, stem-like cells within tumors, commonly designated cancer stem cell (CSC)-like cells also known as tumor-initiating cells (TICs).^1, 2, 3^ CSC-rich tumors are associated with aggressive disease and poor prognosis,^4, 5, 6^ indicating that an understanding of their biology is pertinent to developing effective therapies. However, until recently, it has been unclear what mechanisms control the emergence and maintenance of CSC-like cells.^7, 8^ The current dominant model for CSC has been the pre-existence of a rare cell population with stem cell characteristics within tumors. Recently, a few reports suggest that non-stem cancer cells can spontaneously give rise to a stem-like state, implying stochastic nature of the emergence of CSC-like cells.^1, 9^ Nevertheless, still not much is known about the identity of and functional properties of CSC-like cells in tumor progression. Tumor cell growth in the confined microenvironment causes alterations in metabolic and physicochemical milieu where reciprocal influence between tumor cells and environment would contribute to tumor progression.

The tumor metabolic microenvironment, which is continuously reshaped during tumor progression^10, 11, 12^ can influence adaptive cellular behaviors including dormancy, invasion, and metastasis as well as therapy resistance.^13, 14, 15^ Intriguingly, these acquired phenotypes share characteristics with CSC-like or TICs.^16, 17, 18, 19^ Adaptive behavior of cancer cells in the highly heterogeneous microenvironment^20^ is mediated by induction of changes in gene expression thereby reprogramming signaling pathways.^21, 22^ Furthermore, it was theorized that these emerging adaptive behaviors in cancer might be driven by harsh tumor microenvironmental selective forces.^23^ There are numerous microenvironmental factors that could influence cancer cell behavior, particularly the stem-like characteristics. It is well established and widely accepted that the typical triad of tumor microenvironment consists of hypoxia, nutrient depletion and low pH. Although hypoxia is well studied and known to have a crucial role in driving malignant tumor cell behaviors,^24, 25^ nutrient depletion has not been investigated sufficiently to date in terms of its effect on CSC-like behavior. Furthermore, a recent growing interest in cancer metabolism fueled the rediscovery of oncogenic importance in nutrient utilization and cancer cell biology. As clinical outcome of cancer depends entirely on treatment responsiveness and occurrence of metastasis, which are the contributions of CSCs, we wished to interrogate the emergence of and maintenance of CSC-like cells in the experimental setups mimicking a clinical vignette of nutrient deprivation. We thus show that, in response to chronic metabolic stress (CMS), cancer cells acquire and maintain CSC-like characteristics. This CSC-like transition is mediated through increased Wnt activity induced by metabolic stress. Furthermore, the Wnt pathway can be exploited by cancer cells to execute a CSC-like phenoconversion that facilitates survival under metabolic stress. These results implicate the Wnt pathway as a critical mediator of CSC-like transition of subclone(s) of tumor cells in response to metabolic stress.

## Results

### Phenotypic transition of cancer cells induced by CMS

**Figure 1:**
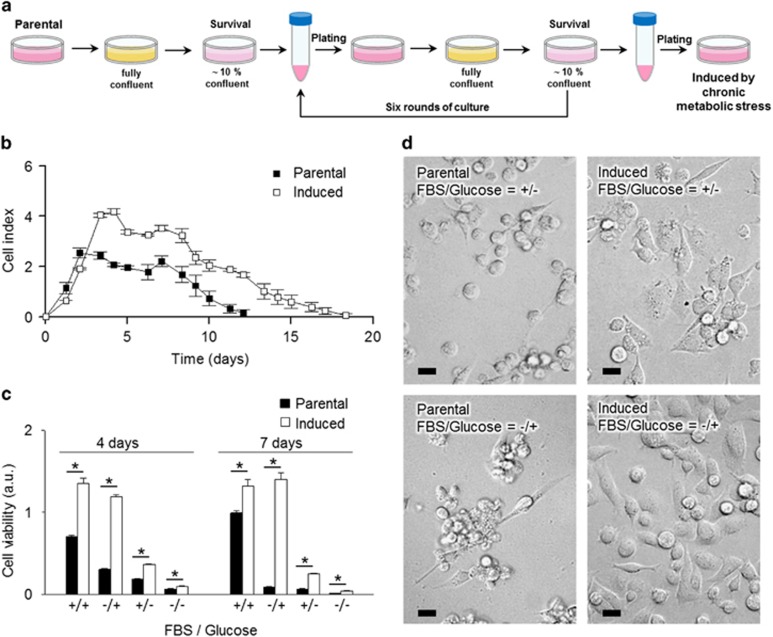
Chronic metabolic stress (CMS) induces an ability to survive acute metabolic stress. (**a**) Schematic illustration of the experimental setup and strategy to derive ‘induced-cells' from parental cancer cells. (**b**) Viability of parental cells and induced cells were determined by real-time cell analyzer (RTCA). Error bars denote the S.E. (*n*=3). (**c**) Cell viability of parental cells and induced cells (day 4 and day 7, respectively) with or without FBS (5%) and glucose. Error bars denote the S.E. (*n*=3). (**d**) Phase-contrast microphotographs showing morphologies of parental and induced cells in the presence (+) or absence (−) of FBS and glucose. Scale bars represent 10 *μ*m. *P<0.01

To investigate the impact of microenvironment-induced metabolic stress on the transition of non-CSC cancer cells into CSC, MDA-MB-231, a claudin low breast cancer cell line, was cultured for several rounds of prolonged periods in culture medium without addition of fresh media to mimic gradual nutrient depletion and CMS. MDA-MB-231 were initially seeded in nutrient-replete culture medium and continued in culture without changing medium until ~90% of the cancer cells died. The remaining viable cells (~10% confluent) were collected and subjected to six rounds in culture of CMS and designated ‘CMS-induced' cells (Figure 1a). Proliferation and viability of the parental and CMS-induced cells were compared using a real-time cell analyzer (RTCA). Upon regular culture condition with complete fresh medium, parental cells proliferated rapidly reaching a plateau by day 3 (Figure 1b and Supplementary Figure S1A). After the plateau, parental cells began to die with >90% of cells dead by day 11. In contrast, CMS-induced cells continued to proliferate until day 5 with an approximate doubling in cell number. Importantly, CMS-induced cells demonstrated extended viability under metabolic stress, as the medium depleted with glucose after 5–7 days, with 90% cell death being delayed by at least a week compared with parental cells. In Supplementary Figure S1B, both parental and CMS-induced cells exhibited proliferation after 3 days from the seeding. On day 11, an increase of subG1 phase in parental cells was observed compared with CMS-induced cells (Supplementary Figure S1C). Moreover, debris from parental cells was remarkably increased compared with CMS-induced cells. These results suggest that the difference in cell number is mainly due to decreased cell death in CMS-induced cells. To define dependence on extracellular nutrients, parental and CMS-induced cells were cultured in nutrient-replete (complete media) and nutrient-deprived conditions (Figures 1c and d). Over time, CMS-induced cells exhibited markedly higher viability than parental cells, which rapidly lost viability under glucose-deprived conditions regardless of presence or absence of fetal bovine serum (FBS; Figures 1c and d). Notably, FBS-deprived parental cells showed a dramatic change in cell fate with most cells undergoing cell death, while CMS-induced cells were insensitive to FBS-deprivation over a 7 day culture period. In the absence of glucose, parental cells were detached from the plate acquiring a spherical shape within 4 days (Figure 1d). In contrast, CMS-induced cells remained attached to the plate maintaining a viability which is a reminiscence of extended survival period in CMS mimicking culture for at least 10 days (Supplementary Figure S1A). These observations suggest that CMS-induced cells gain an ability to survive under prolonged metabolic stress. This difference in survival is not due to decreased cell numbers requiring fewer nutrients, as the number of CMS-induced cells present at each time point was greater than that of parental cells (Supplementary Figure 1B).

### CMS-induced cancer cells exhibit CSC-like properties

**Figure 2:**
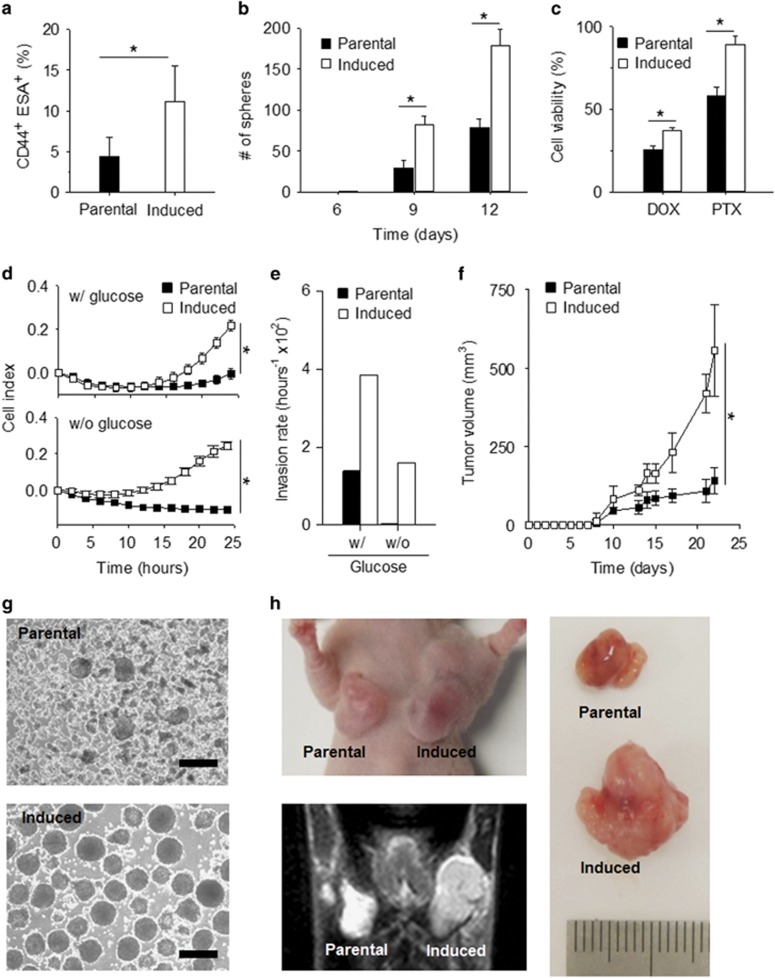
CMS-induced cancer cells acquire CSC-like characteristics. (**a**) Flow cytometry analysis for CD44 and ESA as cancer stem cell markers in parental and CMS-induced cells (MDA-MB-231). (**b**) Mammosphere-forming ability of parental cells and CMS-induced cells was counted every 3 days. (**c**) Cell viability of parental and CMS-induced cells treated with doxorubicin (DOX) or paclitaxel (PTX) at the specified doses for 3 days. (**d**) Invasion potential of parental and CMS-induced cells through matrigel with (w/) or without (w/o) glucose. (**e**) Invasion rates for parental and CMS-induced cells at 24 h on w/ or w/o glucose conditions. (**f**) Tumor-growth curves of orthotopic xenograft mice with parental or CMS-induced cells. Error bars denote the S.E. (*n*=3). *P<0.01. (**g**) Phase-contrast microscopy of mammospheres at day 7. Scale bars, 100 *μ*m. (**h**) Photograph (upper) and MR image (lower) of tumor mass in xenograft mouse with orthotopic implantation of CMS-induced (right) and parental cells (left). Extracted tumor tissues for orthotopic xenograft mouse (right panel)

On the basis of the characteristics of CMS-induced cells under metabolic stress, we determined whether CMS-induced cells would gain stem-like properties. As assessed by flow cytometry, CMS-induced cells exhibited three times higher expression of CD44 and ESA (epithelial-specific antigen)^6^ compared with parental cells (Figure 2a). Consistent with the increase in expression of CSC-associated markers, CMS-induced cells demonstrated a marked increase in mammosphere formation compared with parental cells (Figures 2b and g). As CSC-like cells exhibit drug resistance,^7^ we treated CMS-induced cells with 50 *μ*M of doxorubicin (DOX) and 50 nM of paclitaxel (PTX).^26, 27^ In both cases, CMS-induced cells demonstrated significantly higher viability than parental cells under therapy stress (Figure 2c). Moreover, RTCA analysis combined with matrigel assay revealed that the CMS-induced cells have increased invasive potential compared with parental cells independent of presence or absence of glucose (Figures 2d and e). Finally, to examine tumor-initiating capacity of CMS-induced cells, parental and CMS-induced cells were injected into the right and left thoracic mammary fat pad, respectively, and tumor volumes observed for 23 days. The growth potential of CMS-induced cells was markedly greater than parental cells with an over fivefold difference in tumor volume (Figures 2f and h). More importantly and pertinent to self-renewal capacity of CSC-like properties, CMS-induced cells exhibited greater tumorigenic potentials than parental cells in limiting dilution tumor formation assay (Supplementary Table S1). To further validate whether these stem-like characteristics could be gained under the controlled conditions, parental cells were subjected to glucose-deprived culture and CSC-like cells (Chronic) were retrieved. Taken together, the results indicate that phenotypes associated with ‘stemness' can be induced by metabolic stress *in vitro* and that the effects of metabolic stress are manifest on return to normal culture conditions or in tumor formation *in vivo*.

### CMS-induced cells emerge through stochastic adaptation by cell state transition

**Figure 3:**
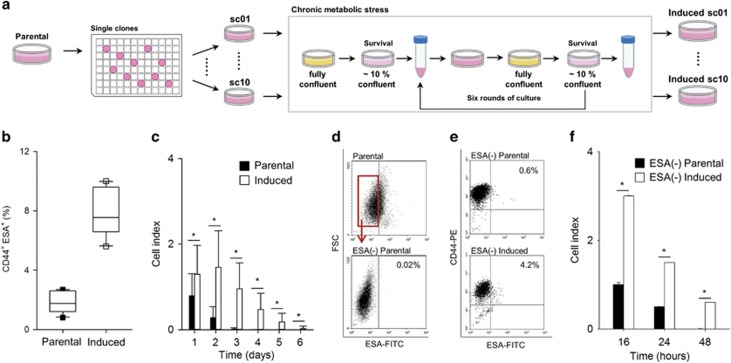
Single clonal (sc)-derived parental cells exposed to CMS convert to CD44^+^ESA^+^ mammary CSC-like state. (**a**) Schematic illustration of the experimental setup and strategy to derive CMS-induced cells from sc parental cells. Ten single parental clones were cultured under CMS to assess clonal variation. (**b**) Box plot displaying CD44 and ESA expression in 10 parental and CMS-induced clones by Flow cytometry analysis. (**c**) Cell index of 10 parental and CMS-induced clones grown in culture without glucose. Error bars denote the S.E. (*n*=10). (**d**) The negative enrichment of ESA-positive cells in parental cells (ESA (−) parental) and Flow cytometry analysis for ESA expression following sorting out ESA-positive cells. (**e**) Flow cytometry analysis for CD44 and ESA expression in CMS-induced cells derived from ESA(−) parental cells (ESA (−) induced). (**f**) Cell index of ESA (−) parental and ESA (−)-induced cells grown in culture without glucose. Error bars denote the S.E. (*n*=3). *P<0.01

To elucidate whether the acquisition of CSC-like properties occurs through selection of pre-existing subclone(s) or stochastic adaptation^1^ of random cells in evolutionary response to microenvironmental CMS, we performed limiting dilution of parental cells in 96 wells and randomly chose 10 single cell-derived clones and repeated the CMS-simulating culture as described in Figure 3a. Of note, all clonally expanded cells recapitulated characteristics of the CMS-induced cells consistent with stochastic adaptation rather than the selection of a pre-existing subpopulation of MDA-MB-231 cells. Each of the CMS-induced clones exhibited higher CSC-associated marker (CD44 and ESA) expression compared with parental clones (Figure 3b and Supplementary Figure S2). Furthermore, in the absence of glucose, the CMS-induced clones demonstrated increased survival (Figure 3c).

As indicated above, CD44^+^ESA^+^ expression level was increased in CMS-induced cells compared with parental cells (Supplementary Figure S3A). Interestingly, CD44 expression was not significantly changed between parental and CMS-induced cells (Supplementary Figure S3B). Rather, the increase in CD44^+^ESA^+^ cells was primarily due to the increased ESA expression that increased from 8.7% in parental cells to 39.3% in CMS-induced cells (Supplementary Figure S3C).

Next, to determine whether the subpopulation of ESA^+^ cells in parental line are responsible for the phenotypic transition induced by CMS, ESA-negative parental cells were collected by fluorescence-activated cell sorting of ESA-positive cells away from the population (Figure 3d). ESA-negative parental cells were then repeatedly exposed to *in vitro* CMS conditions (see Figure 1a). Following CMS selection, of note, the induced cells from ESA-negative parental cells acquired a significant proportion of ESA^+^ subpopulation (Figure 3e) as well as the ability to survive in low glucose conditions (Figure 3f). Next, to elucidate whether the effect observed upon adaptation to metabolic stressing conditions is indeed due to the increased number of CSC-like cells in the population, ESA-positive cells were sorted from parental or induced cells by using a flow cytometry (Supplementary Figure S4A). Subsequently, the sorted ESA-positive cell populations were seeded into normal culture medium or glucose-deprived medium (50 and 200 numbers of cells, respectively). After 2 weeks, as shown in Supplementary Figure S4B and C, there were no significant differences in numbers of formed colonies in both seeding conditions (50 and 200 cells) between parental and induced cells. When identical clonogenic assay for ESA-positive populations was conducted in glucose-deprived medium, both ESA-positive parental and -induced cells formed around 100 colonies and there was no statistical difference (Supplementary Figure S4D). Collectively, these results suggest that CMS-induced cells emerge through stochastic adaptation by dynamic phenotypic state transition of parental cells resulting in an increase of CSC-like cells, which is independent of CSC-associated marker status.

### The ability of metabolic stress to convert cells to CSC-like state is generalizable

To determine whether the metabolic stress could induce CSC-like characteristics in ER(+) breast cancer cells, MCF-7 cells, a representative luminal-type breast cancer cell line was assessed. Indeed, CMS increased the number of CD44^+^ESA^+^ MCF-7 cells, albeit in this case through increasing CD44 expression (Supplementary Figure S5A and B). Furthermore, both colony-formation capacity *in vitro* as well as tumor growth *in vivo* were markedly increased in CMS-induced MCF-7 cells (Supplementary Figure S5C and D).

### Activation of TCF/LEF transcriptional activity is required for transition into CSC-like cell state

**Figure 4:**
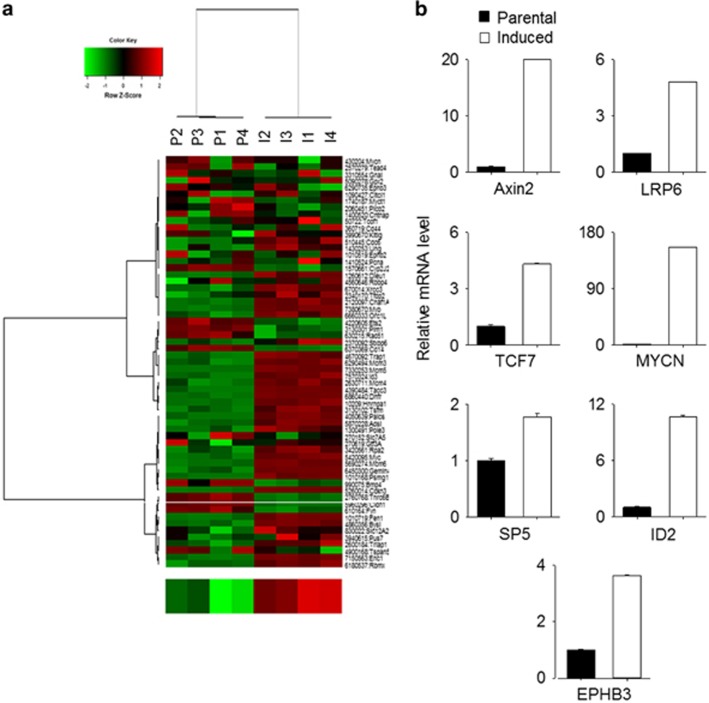
CMS-induced CSC-like cells express Wnt target genes. (**a**) Unsupervised hierarchical clustering of parental and induced cells using a *TCF/LEF* gene signature to compare the Wnt activity (P; parental, I; induced cells). In the heat map, red denotes higher relative expression, whereas green indicates lower relative expression, with degree of color saturation reflecting the magnitude of the log expression signal. The bottom row represents the median log expression value of TCF/LEF target genes. (**b**) Expression levels of mRNAs encoding Axin2, LRP6, TCF7, MYCN, SP5, ID2, and EPHB3 in CMS-induced cells relative to parental cells, respectively, as determined by real-time qRT-PCR. GAPDH mRNA was used as a reference gene. Error bars denote the S.E. (*n*=3)

**Figure 5:**
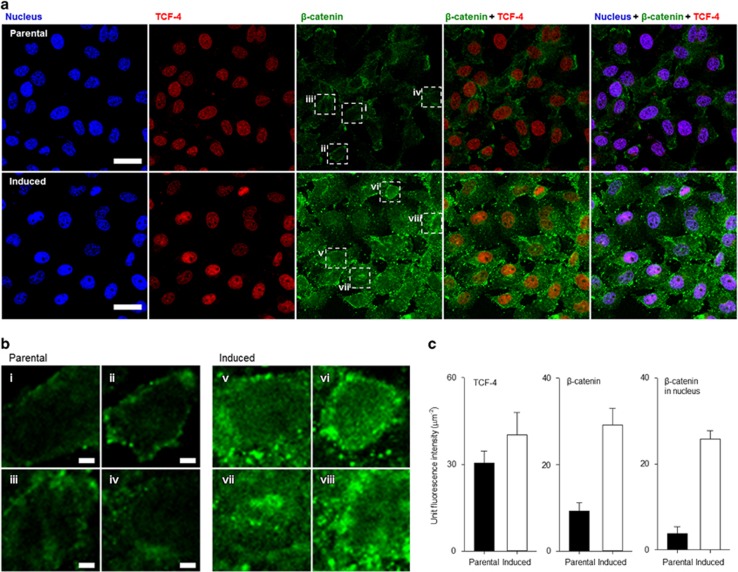
Wnt signaling is activated in CMS-induced cells. (**a**) Confocal microscopic images (scale bars mean 20 *μ*m) and (**b**) magnification images (scale bars mean 2 *μ*m) for parental and induced cells; nucleus (blue), TCF-4 (red), *β*-catenin (green). (**c**) Quantification analysis graphs for TCF-4 and *β*-catenin

**Figure 6:**
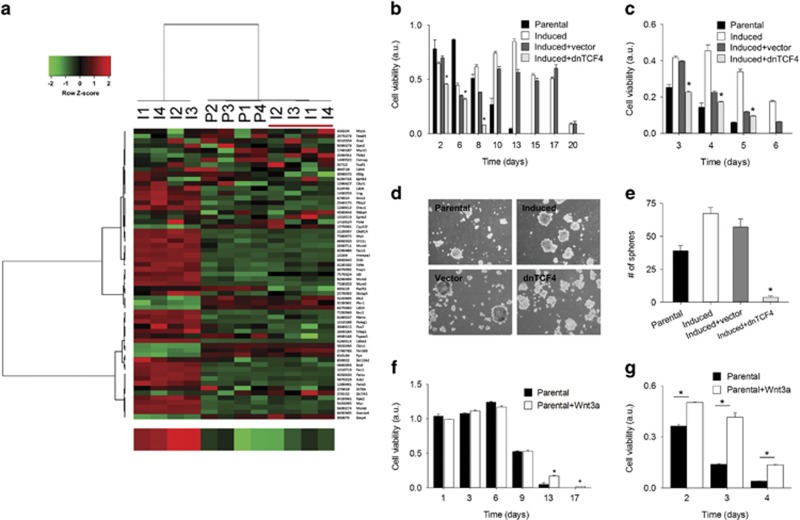
Wnt signaling promotes self-renewal and survival of cancer cells in metabolic stress conditions. (**a**) Unsupervised hierarchical clustering of gene expression using a TCF/LEF transcriptional signature segregates a subset of cells with distinct state (*t*-test, *P*=2.9E−05). P; parental cells, I: CMS-induced cells, underlined; CMS-induced cells followed by refreshment of culture medium. The bottom row denotes the median log-expression value of TCF/LEF target genes. (**b–e**) Cell viability and mammosphere-forming ability after transfection of induced cells with dnTCF4 construct (induced+dnTCF4) or empty vector (induced+vector). Error bars denote the S.E. (*n*=3). (**b**) Long-term survival in regular culture media over 20 days simulating CMS. (**c**) Survival under acute metabolic stress cultivated without glucose. (**d**) Phase-contrast images of mammospheres and (**e**) quantitation of sphere-formation capacity at 12 days. (**f–g**) Cell viability comparison between parental cells in regular culture media (Parental) and those in Wnt3a conditioned media (Parental+Wnt3a). (**f**) Long-term survival with CMS between parental and parental+Wnt3a. Error bars denote the S.E. (*n*=3). (**g**) Survival without glucose between parental and parental+Wnt3a. Error bars denote the S.E. (*n*=3). *P<0.01

Wnt signaling is involved in the maintenance of adult tissue homeostasis as well as in embryonic development.^28^ Furthermore, Wnt activation is linked to breast cancer development.^29^ Recent studies suggest that *β*-catenin/TCF transcriptional machinery coupled with CD44 expression is required for maintaining a CSC-like phenotype and for cancer cell survival during treatment with cytotoxic drugs.^30, 31^ To investigate whether activation of canonical Wnt signaling is involved in CMS-induced CSC-like phenotypic transition, we assessed expression of a gene set involved in Wnt signaling^32, 33^ in CMS-induced cells. In an unsupervised analysis, the expression level of TCF signature was significantly increased in CMS-induced cells (*t*-test, *P*=0.0014, Figure 4a). Independently, increased expression of individual TCF target genes, such as Axin2, LRP6, SP5, TCF7, MYCN, ID2, and EPHB3, were confirmed by real-time PCR (Figure 4b). Furthermore, increase for TCF-4 and *β*-catenin in protein expression level were confirmed by confocal microscopy (Figure 5a). In particular, the localization of *β*-catenin into nucleus site was observed by three-dimensional confocal microscopy (Figures 5b and c and Supplementary Figure S6). Given the ability of CMS-induced cells to proliferate following addition of fresh culture medium, we examined the TCF/LEF signature of CMS-induced cells upon addition of fresh media. Surprisingly, the expression of *TCF/LEF* downstream genes was rapidly attenuated, reaching levels similar to those of parental cells (Figure 6a). Thus, the metabolic conditions induce dynamic reversible Wnt transcriptome expression. Based on these results, we determined whether the Wnt pathway activity is a prerequisite for CMS-mediated CSC-like phenotypic transition by inhibiting Tcf4, a mission-critical transcription factor, function with dominant-negative Tcf4 (dnTcf4).^34^ Strikingly, dnTcf4 decreased survival (Figures 6b and c) and mammosphere formation of CMS-induced cells (Figures 6d and e). Further, to determine whether Wnt activity was sufficient to mimic CMS selection, we investigated the effects of increasing Wnt activity in parental cells. Addition of exogenous Wnt3a to parental cells was sufficient to increase cell survival under both CMS and glucose-depleted conditions (Figures 6f and g). Of note, the difference in survival was only evident at late time points when glucose in the culture medium was depleted. Consistently, the difference was more significant when cells were grown in glucose deprivation conditions. These data support the contention that Wnt signaling is both required and sufficient for CSC-like phenotypic transition under metabolic stress.

## Discussion

Clinically, CSC-rich tumors are associated with aggressive disease and poor prognosis,^5^ indicating that an understanding of their biology is pertinent to developing effective therapies. However, it is unclear that what mechanisms control the maintenance and survival of these TICs. We demonstrate a stochastic and reversible selection of CSC-like cells during metabolic stress. These CMS-induced cells exhibit typical characteristics associated with CSCs including increased survival under metabolic stress, resistance to chemotherapy, increased ability to form tumor spheres, and to seed tumors *in vivo*. The properties were seen following selection both in MDA-MB-231 claudin low and MCF-7 luminal breast cancer cell lines suggesting that the observation is generalizable.

The cell culture system used was designed to mimic natural progression of the tumor microenvironment,^3, 4, 5, 6, 7, 8, 9, 10, 11, 12, 13, 14, 15, 16, 17, 18, 19, 20, 21, 22, 23, 24, 25, 26, 27, 28, 29, 30, 31, 32, 33, 34, 35, 36, 37, 38^ wherein tumor cells compete for space and nutrients. In this setting, unlike conventional standard cell cultures in which cells are cultured in nutrient-replete media before selective pressure arises, evolutionary adaptation is an inevitable consequence.^10, 11^ Given that genetic variation or heterogeneous traits that might exist in the cell lines and limited resources and environmental challenges within the confined culture condition,^8^ fitness tests are spontaneously imposed and subclones with best adaptability will emerge.^39, 40, 41^ One notion is that these ‘subclones' are actually the outcome of evolutionary adaptation of cancer cell population whether purely induced from genetic variation or adapted through non-genetic mechanisms (e.g., epigenetics).^41^ The emergence of adaptive phenotype could be due to either or both processes.^42, 43, 44, 45, 46, 47^ The ability of individual single clones, as well as the ESA (−) parental cells, to recapitulate the selection process suggest an adaptive mechanism such as metabolic-induced transcriptional reprogramming rather than selection of pre-existing subclones. These experiments indicate that the CD44/ESA marker-defined CSC-like populations might emerge through an adaptive phenoconversion rather than selection of pre-existing CD44^+^ESA^+^ clones.

One potential caveat of this interpretation is that the cell lines used underwent a ‘selection process' either in adaptation to cell culture or during prolonged cell culture that resulted in the acquisition of CMS-induced CSC-like characteristics that are not present in the original tumor. This seems unlikely given the observation that the metabolic state of tumors reflects that of nutrient (and oxygen) deprivation.

On the contrary to the common notion that CSC-targeted therapy can eliminate the root of cancer, the reversible nature of the phenotype conversion between non-stem cancer cells and CSC-like cells have clinical implications that anti-cancer therapies exclusively targeting CSC-like cells may not be sufficient to completely eradicate tumors.

Wnt signaling is essential during embryo development and in the maintenance of adult tissue homeostasis through the regulation of adult stem cell function.^28, 48^ Therefore, Wnt pathway components are natural candidates as genetic factors that predispose to or trigger cancer progression.^34, 49^ A number of breast cancer cell lines contain a small population of cells that mimic CSC behaviors.^17^ Similar to primary breast cancers, cell line-derived TICs are enriched in cells with the CD44^+^/CD24^−/low^/ESA^+^ phenotype.^26, 50^ Although the Wnt pathway may be a common element in regulating stem cell renewal and maintenance in a variety of systems,^51^ how this ability is exploited by cancer remains unclear. It is also tempting to speculate that aberrant Wnt signaling is involved in the generation of CSC-like cells from cancer cells without CSC properties.^31, 52^ Based on our data, it is reasonable to argue that the Wnt signaling pathway is hijacked to maintain survival during selective pressure mediated by metabolic stress in the microenvironment. Regardless of the mechanism of the increased Wnt activity in CMS-induced cells, it is clear that transcriptional reprogramming favors the expression of genes related to Wnt signaling to promote the transition to a stem-like state during CMS.

## Conclusions

In conclusion, we demonstrate that selective pressure from CMS drives the stochastic state transition of non-CSC cells to CSC-like cells, which is dependent on Wnt pathway. Notably, Wnt-mediated enhanced survival of CMS-induced cells is distinct under glucose deprivation conditions. Thus, Wnt signaling represents a potential target to both prevent the emergence and reverse a CSC-like phenotype under metabolic stress during tumor progression.

## Materials and Methods

### Cell culture and generation of induced cell lines

Human breast cancer cell lines, MDA-MB-231 parental cells and CMS-induced MDA-MB-231 cells, from MDA-MB-231 parental cells were cultured in RPMI-1640 with 5% FBS (Gibco, Carlsbad, CA, USA) in a humidified incubator containing 5% CO_2_ at 37 °C. MDA-MB-231 cells were cultured without refreshment of culture medium and recovered for several rounds. Briefly, cancer cells were initially seeded in a usual culture medium and continued in culture without changing medium until 90% of cancer cells died. This process was designated as *in vitro* CMS culture. The remaining viable cells were collected and put in another round of CMS culture conditions for several rounds.

### Constructs and transfections

Transfection into induced cells were carried out using 1.6 *μ*g pPGS-neo or pPGS-dnTCF4 with 80 *μ*g in 100 *μ*l of Opti-MEM (Gibco). Mix 4 *μ*l Lipofectamine 2000 in 100 *μ*l of Opti-MEM. After the 5 min incubation, combine the mixture. Mix gently and incubate for 25 min at room temperature. Then change the growth medium (1 ml) and add the complexes (200 *μ*l) to each well. After 4–6 h, medium may be changed. Subsequent selection of the bulk cell population in G418 (Sigma-Aldrich, St. Louis, MO, USA) at an initial concentration of 1 mg/ml. After 48 h, the G418 concentration was reduced to 0.75 mg/ml. After 1 week, the G418 concentration was further reduced to 250 mg/ml and the expression of the transferred genes was confirmed.

### Chemotherapy treatment

Cells were split and 1 × 10^4^ cells were treated the following day with a 1 : 100 dilution of 0.1 M DOX (Sigma-Aldrich) in dimethyl sulfoxide (DMSO, Sigma-Aldrich) for a final concentration of 50 *μ*M DOX, or 1 *μ*M PTX (Sigma-Aldrich) in DMSO for a final concentration of 50 nM. Placebo control plates received 1 : 100 dilution of DMSO. Cells were analyzed at 3 days.

### Viability and cell proliferation assay

Cells were collected after each induced condition and dissociated into single-cell suspension by trypsin/EDTA. For cell proliferation assay, 1 × 10^4^ cells were seeded in triplicate wells per cell line per time point in 96-well tissue culture plates. The viability of cells were evaluated by a colorimetric assay, based on the cellular reduction of 3-(4,5-dimethylthiazoly-2)-2,5-diphenyltetrazolium bromide (MTT; Cell Proliferation Kit I, Roche, Mannheim, Germany) in metabolically active cells. In a typical cell viability experiment, cells were seeded into 96-microwell plates and incubated at 37 °C. The cells were incubated with fresh medium (100 *μ*l) containing Parental or induced cells at 37 °C. After the incubation, the yellow MTT solution was treated, and the formed formazan crystals were solubilized with 10% SDS in 0.01 M HCl. Then the absorbance of the resulting colored solution was measured at 584 nm and at 650 nm as a reference using a microplate spectrophotometer (Synergy H4, BioTek, Winooski, VT, USA). Cell viability was determined from the intensity ratio of treated to non-treated control cells and shown as an average±S.D. (*n*=3).

### Clonogenic assay for ESA-positive cell populations

ESA-positive cell populations, stained by fluorescein-conjugated anti-mouse ESA antibody, were sorted from MDA-MB-231 parental or -induced cells by using a flow cytometry. Subsequently, the sorted ESA-positive cell populations were seeded in regular culture medium or glucose-deprived medium (50 and 200 of cells, respectively). After 2 weeks, the obtained colonies are fixed and stained (Clonogenic assay kit, BioPioneer, San Diego, CA, USA). When the clonogenic assay for ESA-positive cell populations was conducted in glucose-deprived medium, 2000 of ESA-positive cells (parental or induced) were plated and colonies were assessed after 2 weeks from the seeding.

### Immunofluorescence using confocal microscopy

For visualization of TCF-4 and *β*-catenin expression level, MDA-MB-231 cells (parental and induced cells) were plated at a cell density of 5 × 10^4^ in a flat bottom four-well plate with coverslips and allowed to adhere overnight. The following day, the cells were then fixed for 30 min in phosphate-buffered saline (PBS) supplemented with 4% formaldehyde. Subsequently, the cells were washed three times with PBS and permeabilized with 0.5% Triton X-100 made in PBS solution for 15 min. The cells were washed three times with PBS+0.1% bovine serum albumin (BSA) and incubated for 30 min for blocking. The cells were incubated for 30 min at 22 °C with anti-*β*-catenin mouse antibodies and anti-TCF-4 rabbit antibodies at a 1 : 200 dilution in the wash buffer. After primary serum incubation, the cells were washed three times with PBS+0.1% BSA and incubated for 30 min with goat anti-mouse IgG Antibody, (H+L) fluorescein isothiocyanate (FITC; Cat. AP124F, Millipore, Billerica, MA, USA) and Anti-rabbit IgG (H+L), F(ab')2 fragment (phycoerythrin, PE; Cat. 8885 S, Cell signaling) at 1 : 200 and 1 : 500 dilution for 30 min at 22C. Finally, the cells were washed three times with PBS, then 10 minutes before analyzing the plate a final concentration of 5 mg/ml of Hoeches33342 was added to stain the nucleus. The images were captured by using an confocal microscope (LSM-700, Carl Zeiss, Jena, Germany) and the ZEN software (ver. 5,5,0,375, Carl Zeiss), which was designed for acquisition and processing of confocal images.

### Single clonal assay

Proliferating cells in parental cell dispersed in RPMI-1640 with 5% FBS medium at <10 cells/ml. Single clonal cells were cultured in RPMI-1640 with 5% FBS each 96-well plates 1–2 weeks after seeding. Proliferating cells in 96-well plates were transferred successively to 12- and 6-well plates in the presence of RPMI-1640 with 5% FBS medium. Then, the expanded 10 clones were exposed to CMS. Among them, parental clones and induced clones were gathered and analyzed.

### Wnt3A-conditioned medium experiments

MC3T3-E1 osteoblastic cells, used in the *in vitro* mechanical loading experiments, were cultured in *α*MEM supplemented with 10% heat inactivated FBS, 1% glutamax, and 1% penicillin/streptomycin. Wnt3A-conditioned media was obtained from an overexpressing Wnt3A stable murine L-cell line (ATCC, Manassas, VA, USA) that was maintained in Dulbecco's modified Eagle's medium supplemented with 10% FBS, 1% l-glutamine, and 0.4 mg/ml Geneticin. To obtain Wnt3A-conditioned media, cells were seeded into 100 mm dishes and cultured for 4 days in growth medium without Geneticin, the medium was removed and sterile filtered, and fresh medium was added to the plates and cultured for an additional 3 days. The medium was then removed and sterile filtered, and combined with the initial batch of cultured media.

### Mammosphere assay

1 × 10^5^ cells/ml were seeded in a 6-well ultra-low adhesion plates (Corning, Lowell, MA, USA) in DMEM/F12 (Lonza, Lowell, MA, USA) with 5% FBS medium supplemented with 10 ng/ml EGF, 10 *μ*g/ml insulin, and 1 *μ*g/ml hydrocortisone (Gibco). Two microlitres of medium per well were added every 2 days. The number of spheres for each well was evaluated from 3 to 12 days of culture. Pictures were taken to assess the ratio of spheres to aggregates of cells.

### Fluorescence-activated cell sorting

The expression of CD44 and EpCAM on cells was determined by flow cytometry. Single-cell suspensions from 500 000 cells were collected, washed three times with blocking buffer (0.2% FBS and 0.02% sodium azide in PBS (pH 7.4) to prevent non-specific binding of antibody, and then incubated with FITC-conjugated anti-mouse EpCAM and PE-conjugated anti-human CD44 (BD Biosciences, Bedford, MA, USA) for 30 min at 4 °C. Cells were resuspended in 400 *μ*l of 4% paraformaldehyde solution and stored at 4 °C prior to flow cytometry. For the quantification of DNA content (cell cycle profiling), cells were collected and stained with 1 *μ*g/ml propidium iodide (which only incorporates into dead cells, Sigma-Aldrich), upon staining, cells were incubated for 30 min at 37 °C. Unbound antibody was washed off and cells were analyzed no longer than 1 h post staining on a BD FACScalibur (Becton Dickinson, San Jose, CA, USA).

### Microarray experiment and data analysis

Total RNA was isolated from cells harvested after each treatment by using mirVana miRNA Isolation Kit (Ambion Inc., Austin, TX, USA) according to manufacturer's protocol. Biotin-labeled cRNA was prepared by using the Illumina TotalPrep RNA Ampliification Kit (Ambion Inc.). Total RNA (500 ng) was used for the synthesis of cDNA followed by amplification and biotin labeling as recommended by the manufacturer. Biotinylated cRNA (1.5 *μ*g) per sample was hybridized to Illumina Human-6 BeadChip v.2 microarray and signals were developed by Amersham fluorolink streptavidin-Cy3 (GE Healthcare Bio-Sciences, Little Chalfont, UK). Data were analyzed using Illumina Bead Studio v3.0 after scanning with Illumina bead Array Reader confocal scanner (BeadStation 500GXDW; Illumina Inc., San Diego, CA, USA). All statistical analysis was performed using R 2.3.0 and BRB Arraytools Version 3.5 (http://linus.nci.nih.gov./BRB-ArrayTools.html) with quantile normalization. Transcriptional profiling of TCF-responsive genes was described previously.^33^ Briefly, genes regulated by TCF/LEF were obtained from independently published results^32^ and matched to the corresponding Illumina probes. The TCF/LEF signature consisted of 74 genes that were responsive to dominant-negative TCF-4 (twofold cut-off) in colon cancer cells. For an unsupervised hierarchical cluster analysis of TCF/LEF, Ward linkage method was used together with the Pearson distance for both sample and gene clustering. The statistical significances of the association between the hierarchical clusters of TCF/LEF genes were determined by two-tailed Fisher's exact test.

To minimize the effect of variation from non-biological factors, the values of each sample were normalized using a quantile normalization method. Random-variance *t*-test was applied for the calculation of significance of each gene in the comparison of two classes and one-way ANOVA was applied for the evaluation of significance in multi-group comparison. Cluster analysis was performed with Cluster and Treeview (http://rana.lbl.gov/EigenSoftware.htm). For cluster analysis, log_2_ transformed data were centered to mean values of each gene's expression. Gene Set Enrichment Analysis was performed against GO (Gene Ontology) of Biological Process and nine Kolmogorov–Smirnov statistic was applied for the evaluation of statistical significance of each GO category.

### Quantitative real-time RT-PCR

RNA was extracted using the RNA isolation Kit (Ambion, Foster City, CA, USA), total RNA (1 *μ*g) was reverse transcribed to cDNA by using the SuperScript III First-Strand Synthesis System (Invitrogen, Carlsbad, CA, USA) according to the manufacturer's protocol. The primer sequences used for the reactions are in Supplementary Figure S7. RT-PCR was performed in LC480 (Roche) Sequence Detection System. GAPDH was used as the reference gene.

### RTCA proliferation, invasion, and migration assays

RTCA (xCELLigence Roche, Penzberg, Germany) proliferation, invasion, and migration assay measures the effect of any perturbations in a label-free real-time setting. For RTCA proliferation experiments, 1 × 10^4^ cells were in serum medium for 20 days and seeded in RTCA E-16 plates. For RTCA invasion or migration experiments, 4 × 10^4^ cells were then starved in serum-free medium for 24 h and seeded in RTCA CIM-16 plates in serum-free medium. Full-growth medium was used as a chemoattractant in the lower chamber. Measurements by cell index were performed in a time-resolved manner using the RTCA device. For invasion assays, the CIM-16 plates were initially coated with Matrigel (BD Biosciences) diluted in serum-free medium at a ratio of 1 : 20. Parental and induced cells were stimulated to invade in the presence or absence of glucose.

### Animal protocol

Female nude mice (BALB/c-Slc, 4–6 weeks) were used for *in vivo* tumor-growth studies. All experiments were conducted with the approval of the Association for Assessment and Accreditation of Laboratory Animal Care International. All of the *in vivo* studies were carried out under approved institutional experimental animal care and use protocols. For orthotopic injections, parental and induced cells were resuspended at 1 × 10^6^ cells/100 *μ*l in 1 : 1 (v/v) media and Matrigel (BD Biosciences, San Jose, CA, USA) and injected into mammary fat pad of 4-week-old athymic nude mice, respectively. Mice were weighed, and tumor measurements were taken in three coordinates using digital calipers two to three times weekly. Tumor measurements were converted to tumor volume using the formula L × S^2^/2 (where L, longest diameter; S, shortest diameter). At sacrifice, mice were weighed, and tumors excised and assessed histologically for verification of tumor growth. Statistical significance was determined using Student's *t*-test.

### Animal MR imaging

MRI of the xenograft mice model was performed with a 3 T clinical MR imager (Philips Medical Systems, Best, The Netherlands). For T2-weighted MRI at 3 T, the following parameters were adopted: point resolution=185 × 185 *μ*m, section thickness=0.6 mm, TE=80 ms, TR=5142 ms, and number of acquisitions=1.

### Statistical analysis

*In vitro* results are expressed as mean±S.D. and *in vivo* results are expressed as mean±S.E. Student's *t*-test was performed to determine statistically significant differences between groups, and *P*-values (<0.01 or 0.05) were considered statistically significant.

## Abbreviations

CSC: cancer stem cell
CMS: chronic metabolic stress
TICs: tumor-initiating cells
RTCA: real-time cell analyzer
ESA: epithelial-specific antigen
DOX: doxorubicin
PTX: paclitaxel
