# Supplementary material for: Metabolic stress induces a Wnt-dependent cancer stem cell-like state transition
Source: Cell Death Dis. 2015 Jul 2;6(7):e1805–. doi: 10.1038/cddis.2015.171 (PMC4650724; doi:10.1038/cddis.2015.171)
Supplement: Supplementary Information [file cddis2015171x1.doc]

***Supporting Information***

**Metabolic stress induces a Wnt-dependent cancer stem cell-like state transition**

*Eugene Lee^1, 2^, Jaemoon Yang^1,3^, Minhee Ku^1,4^, Nam Hee Kim^5^, Yeonji Park^1^, Chang Bum Park^5^, Jin-Suck Suh^1,3,6^, Eun Sung Park^3^, Jong In Yook^5^, Gordon B Mills^7^, Yong-Min Huh^1,3,6^ and Jae-Ho Cheong^6,8,9^*

^1^Department of Radiology, Yonsei University College of Medicine, 120-752, Republic of Korea, ^2^Nanomedical National Core Research Center, Yonsei University, 120-749, Republic of Korea, ^3^Yonsei-KRIBB Medical Convergence Research Institute, Yonsei University Health System, Seoul, Korea, ^4^Brain Korea 21 PLUS Project for Medical Science, Yonsei University, ^5^Department of Oral pathology, Oral Cancer Research Institute, Yonsei University College of Dentistry, 120-752, Republic of Korea, ^6^Severance Biomedical Science Institute (SBSI), 120-752, Republic of Korea, ^7^Department of Systems Biology, MD Anderson Cancer Center, Houston, Tx, ^8^Department of Surgery, Yonsei University College of Medicine, 120-752, Republic of Korea, ^9^Department of Biochemistry & Molecular Biology, Yonsei University College of Medicine, 120-752, Republic of Korea

*Correspondence to Yong-Min Huh or Jae-Ho Cheong

E. Lee and J. Yang contributed equally to this work.


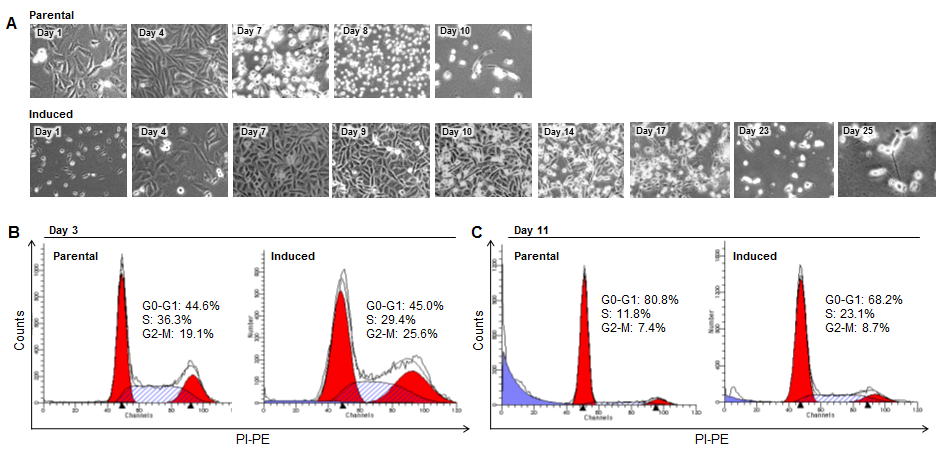


**Figure S1** CMS-induced cells demonstrate increased survival in long-term culture. (a) Phase-contrast microscopy showing morphology of cultured parental and CMS-induced cells. Cell cycle assays of parental and CMS-induced cells were determined by FACS staining with propidium iodide; (b) day 3 and (c) day 11.


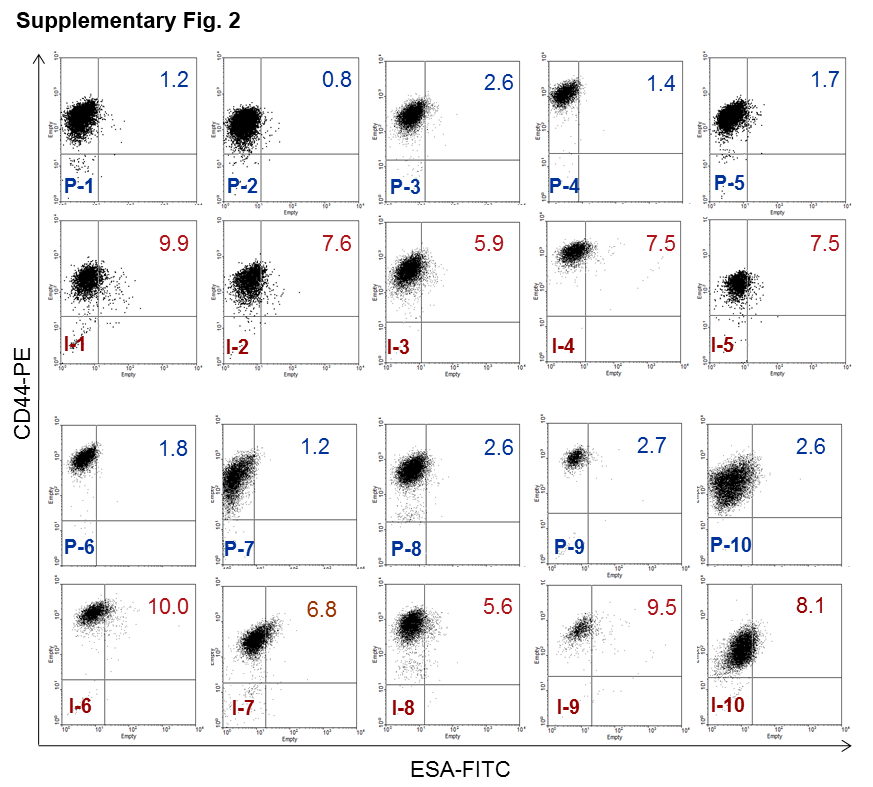


**Figure S2** Stem-like transition of monoclonal parental cells by CMS. (a) Schematic illustration of the experimental setup and strategy to derive CMS-induced cells from single clonal parental cells. (b) Flow cytometry profiles for CD44 and ESA of monoclonal parental cells and CMS-induced cells derived from those cells.


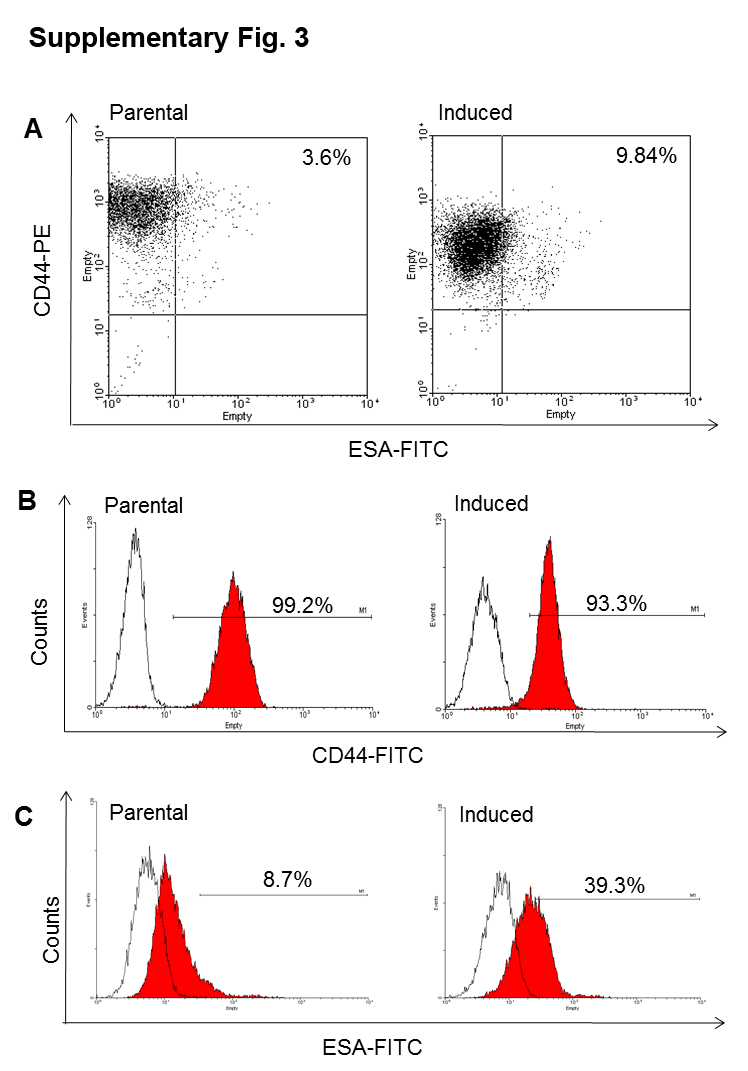


**Figure S3** CMS-induced cells exhibit CD44^+^ ESA^+^ mammary CSC-like state. (a) Flow cytometry profiles for CD44 and ESA of parental and CMS-induced cells. (b) Flow cytometry profiles for CD44 of parental and CMS-induced cells. (c) Flow cytometry profiles for ESA of parental and CMS-induced cells.


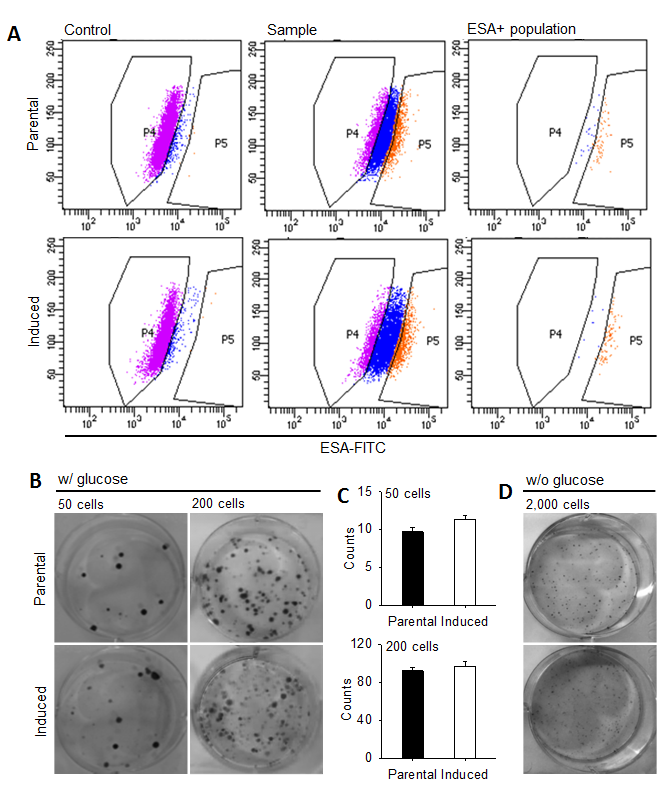

**Figure S4.** (a) The enrichment of ESA-positive cells in parental (upper) and induced (lower) cells by flow cytometry sorting. (b) Photographs for representative colonies of parental (upper) and induced (lower) cells for ESA-positive populations in glucose-replete medium condition; seeding condition: 50 cells (left column) and 200 cells (right column). (c) Quantification of the clonogenic assays from (b). (d) Photographs for representative colonies of parental (upper) and induced (lower) cells for ESA-positive populations under glucose-deprived medium (seeding condition: 2000 cells).


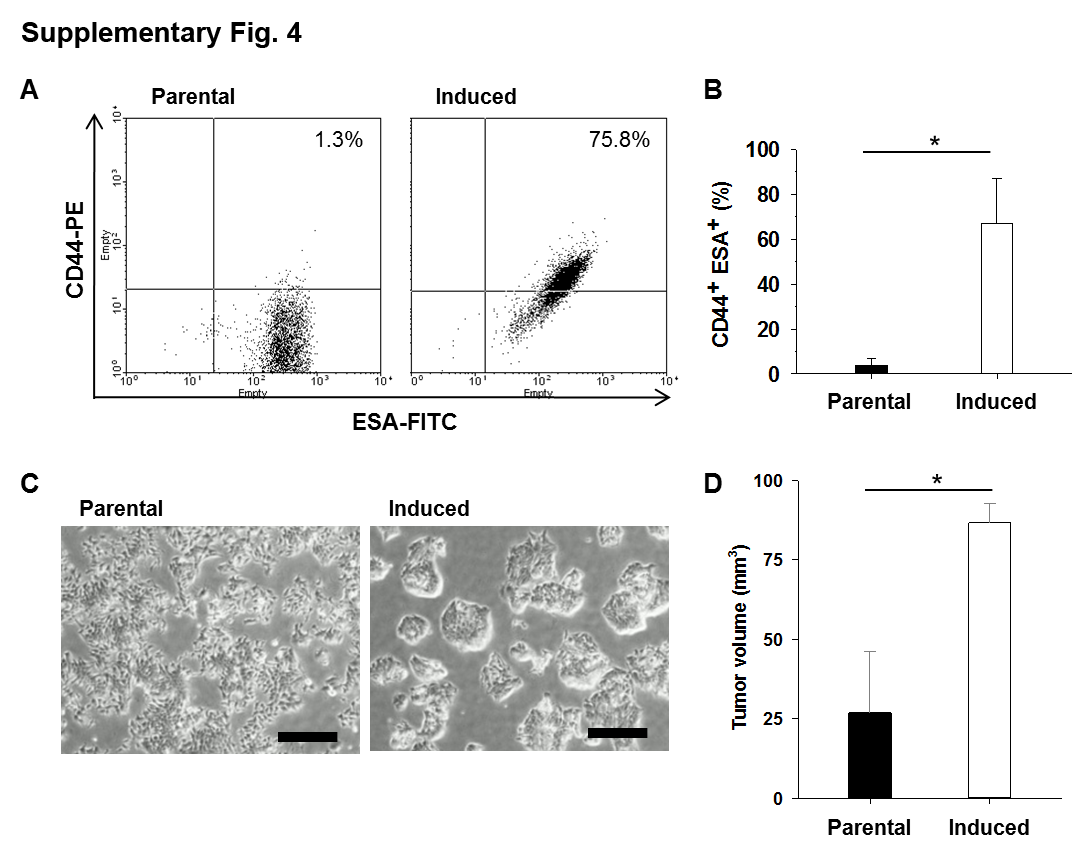


**Figure S5** CMS-induced cells derived from parental MCF-7 cells share CSC-like characteristics. (a) Flow cytometry analysis for CD44 and ESA as cancer stem cell markers in parental and CMS-induced MCF-7 cells. (b) Flow cytometry averages for CD44 and ESA as cancer stem cell markers in parental and CMS-induced MCF-7 cells. (c) Phase-contrast bright field image of parental and CMS-induced MCF-7 cells at day 3. (d) Tumor volume of mice xenograft with parental or CMS-induced MCF-7 cells. Error bars denote the standard error (n = 3). *p<0.01.


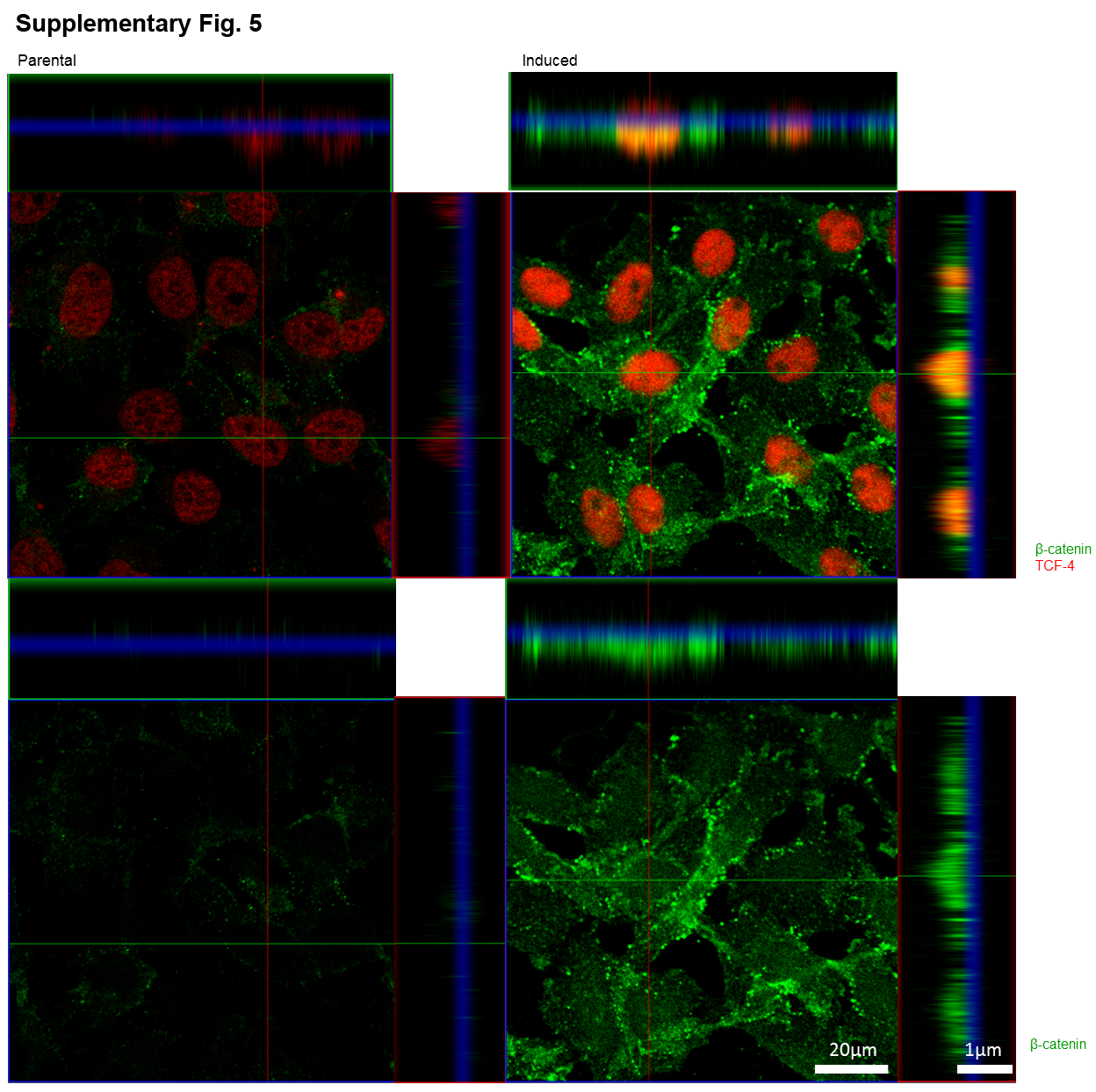


**Figure S6** 3-dimensional confocal microscopic images for parental (left) and induced cells (right); β-catenin (green) and TCF-4 (red).


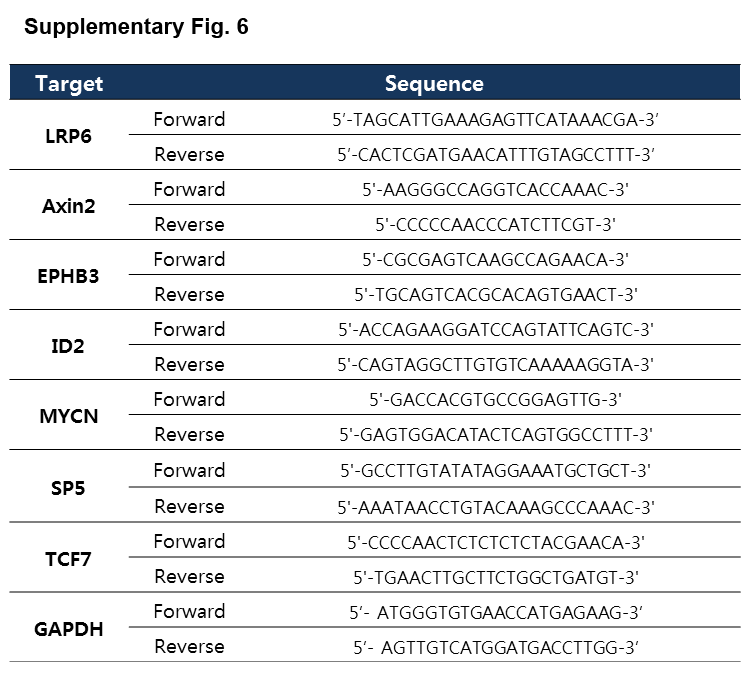


**Figure S7** The primer sequences used for quantitative real-time PCR.

**Table S1** Limiting dilution tumor formation of parental and induced cancer cells.

**
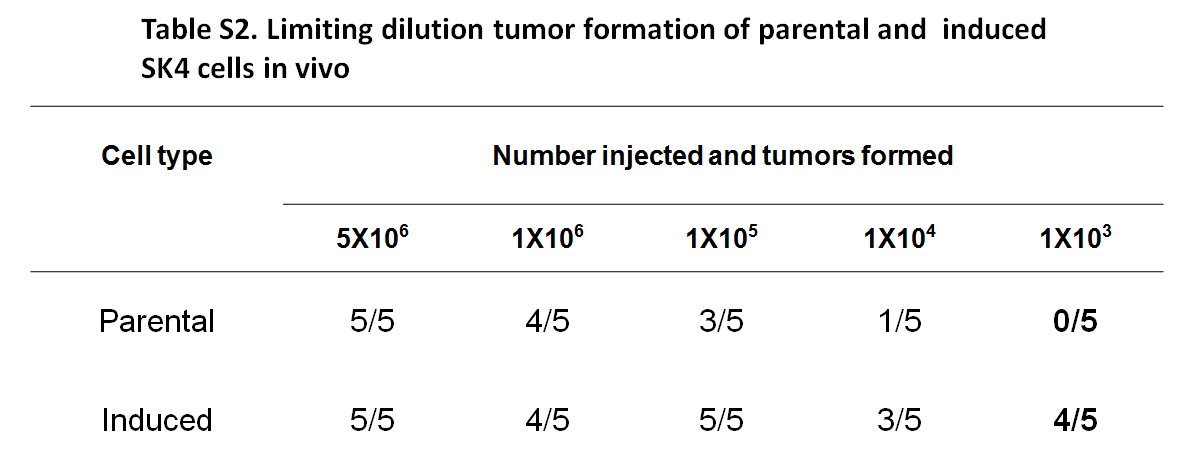
**
