# Supplementary figures and images for: Metabolic stress induces a Wnt-dependent cancer stem cell-like state transition
Source: Cell Death Dis. 2015 Jul 2;6(7):e1805–. doi: 10.1038/cddis.2015.171 (PMC4650724; doi:10.1038/cddis.2015.171)

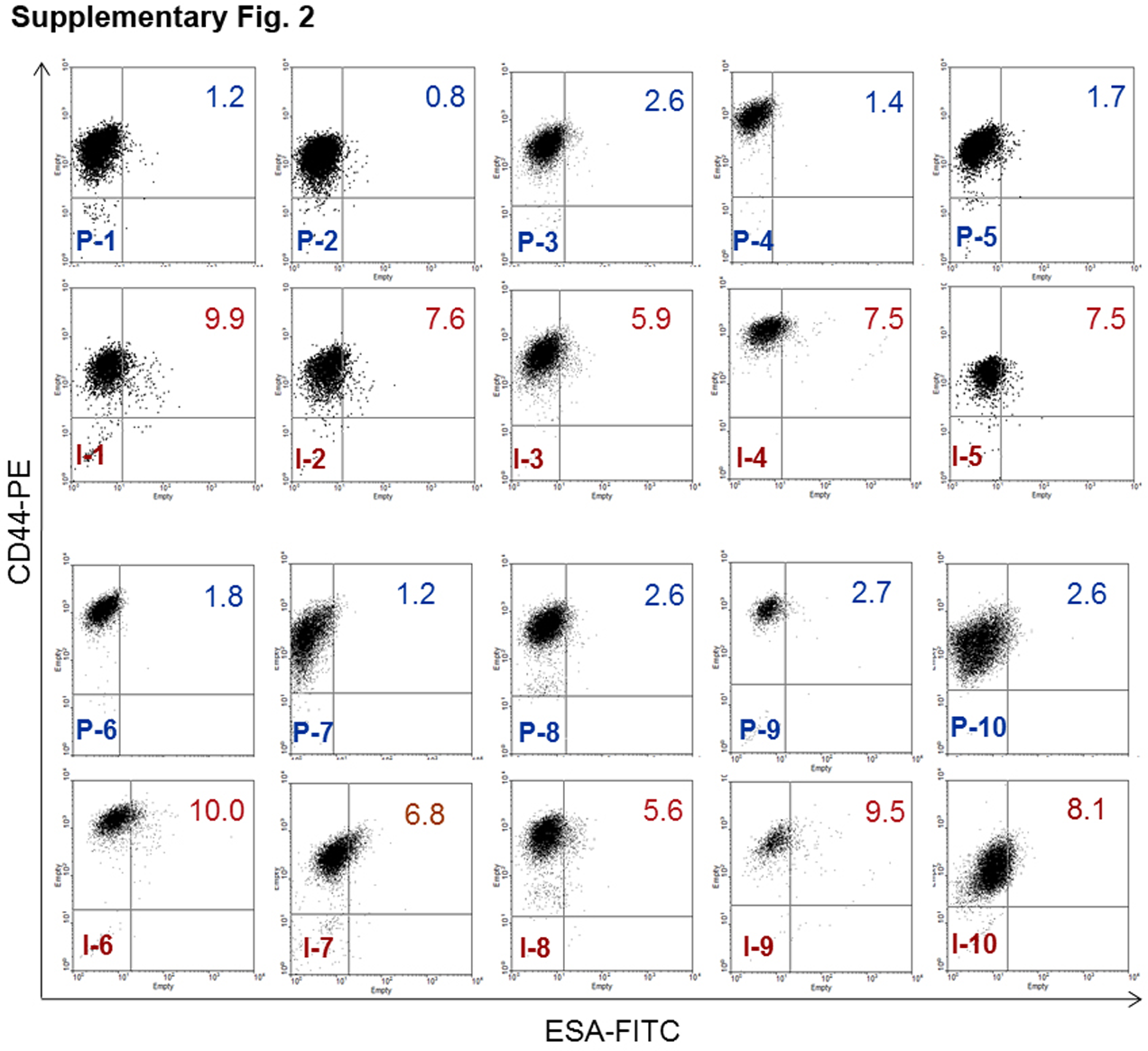

Supplement: Supplementary Figure S2 [file cddis2015171x3.tif]

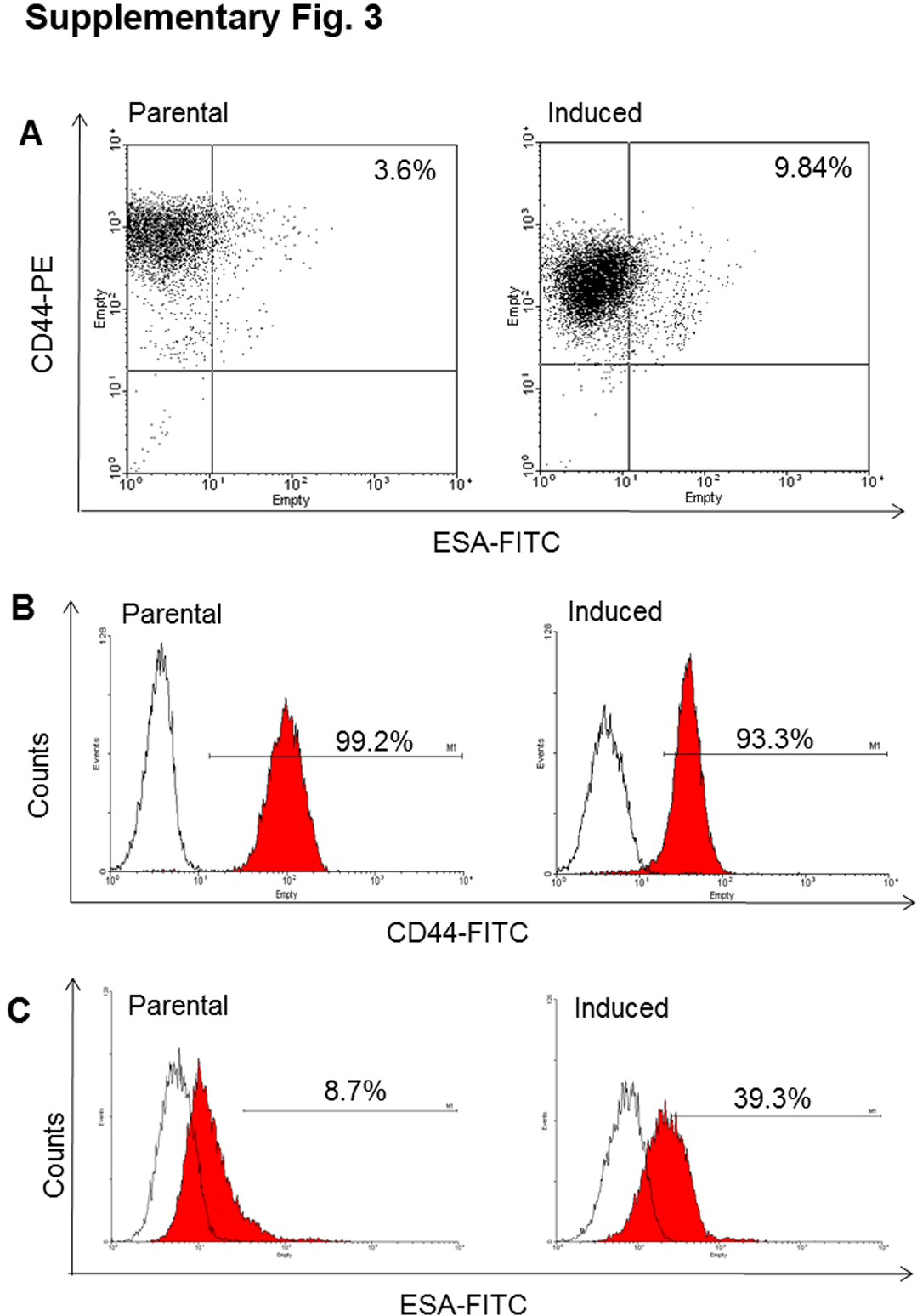

Supplement: Supplementary Figure S3 [file cddis2015171x4.tif]

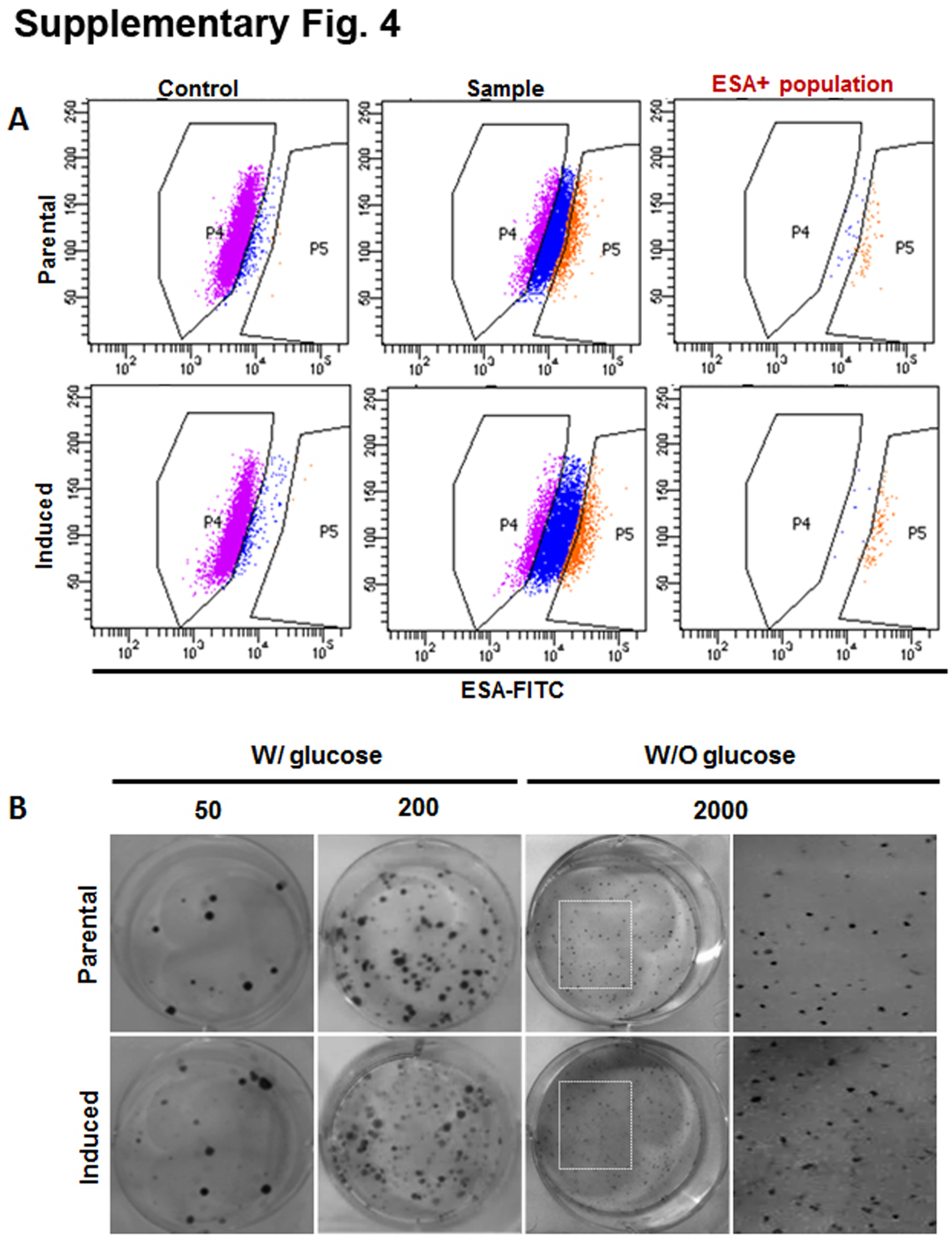

Supplement: Supplementary Figure S4 [file cddis2015171x5.tif]

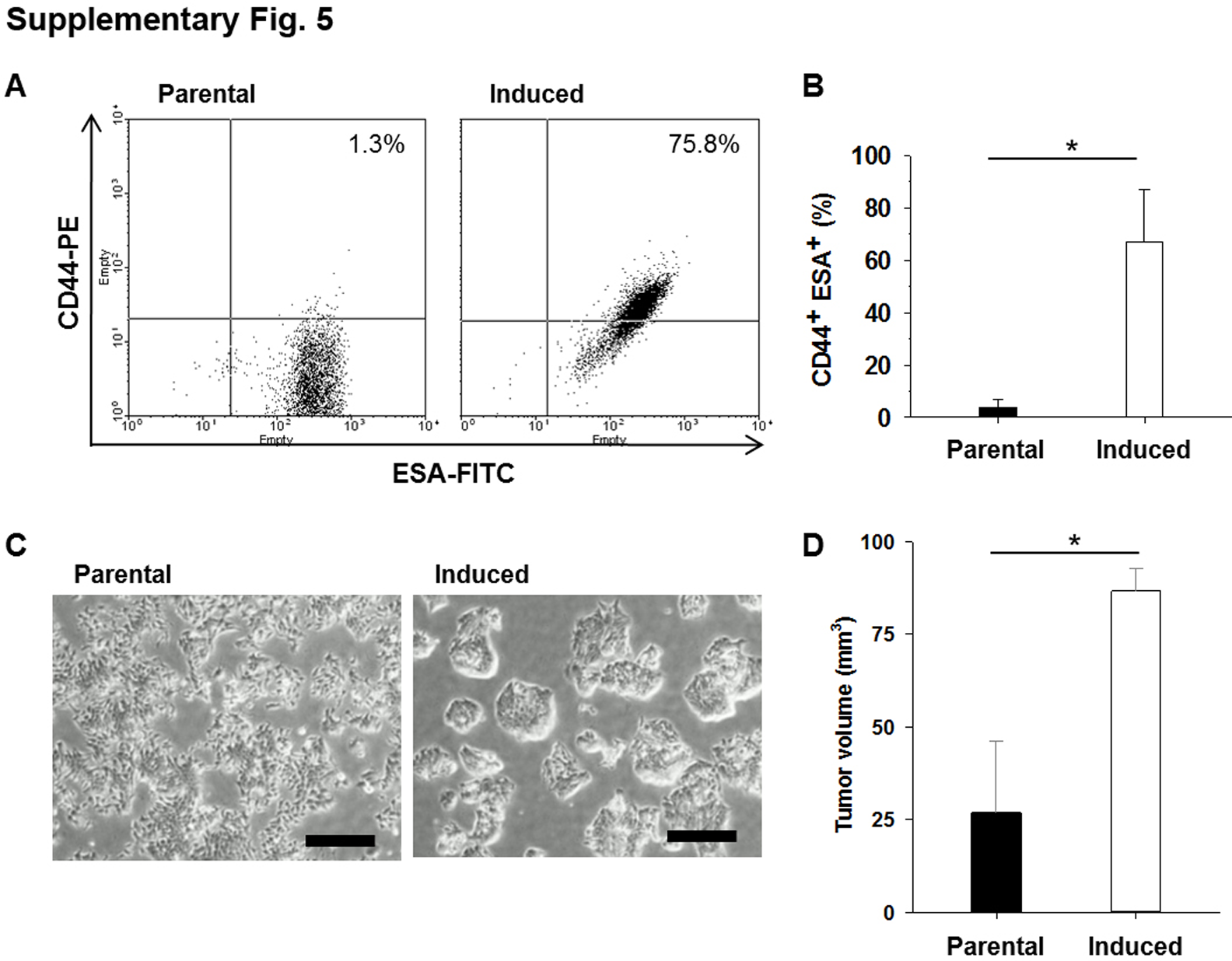

Supplement: Supplementary Figure S5 [file cddis2015171x6.tif]

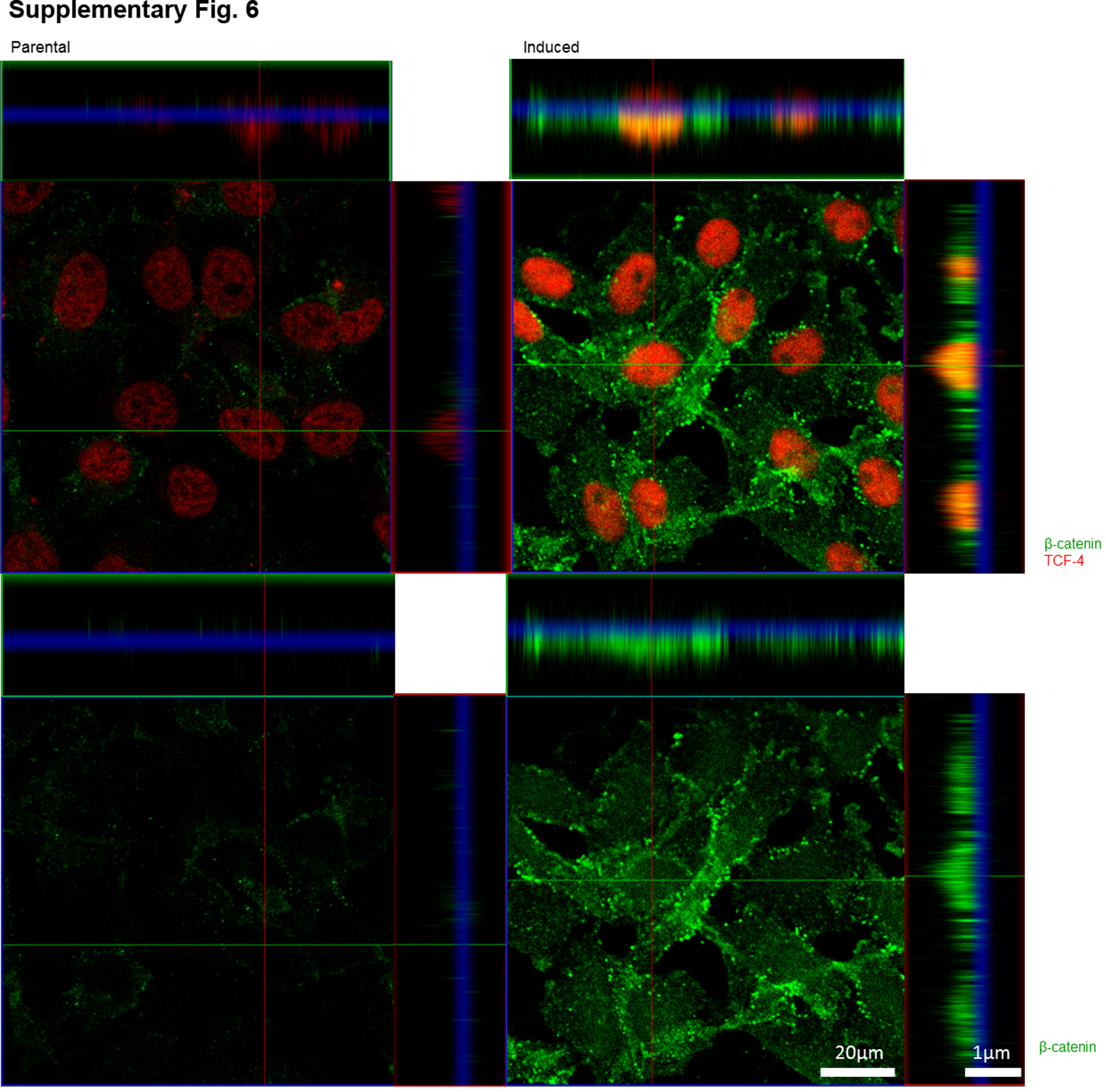

Supplement: Supplementary Figure S6 [file cddis2015171x7.tif]

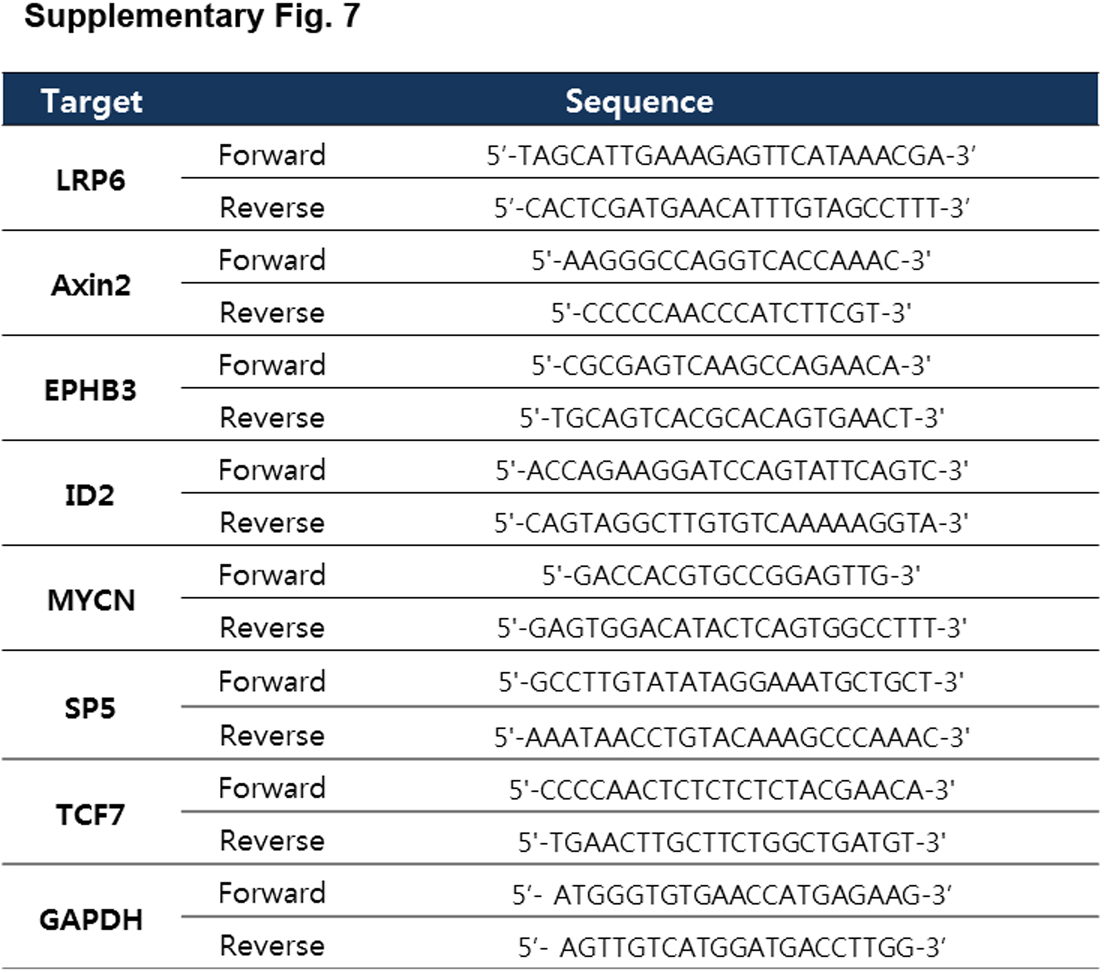

Supplement: Supplementary Figure S7 [file cddis2015171x8.tif]

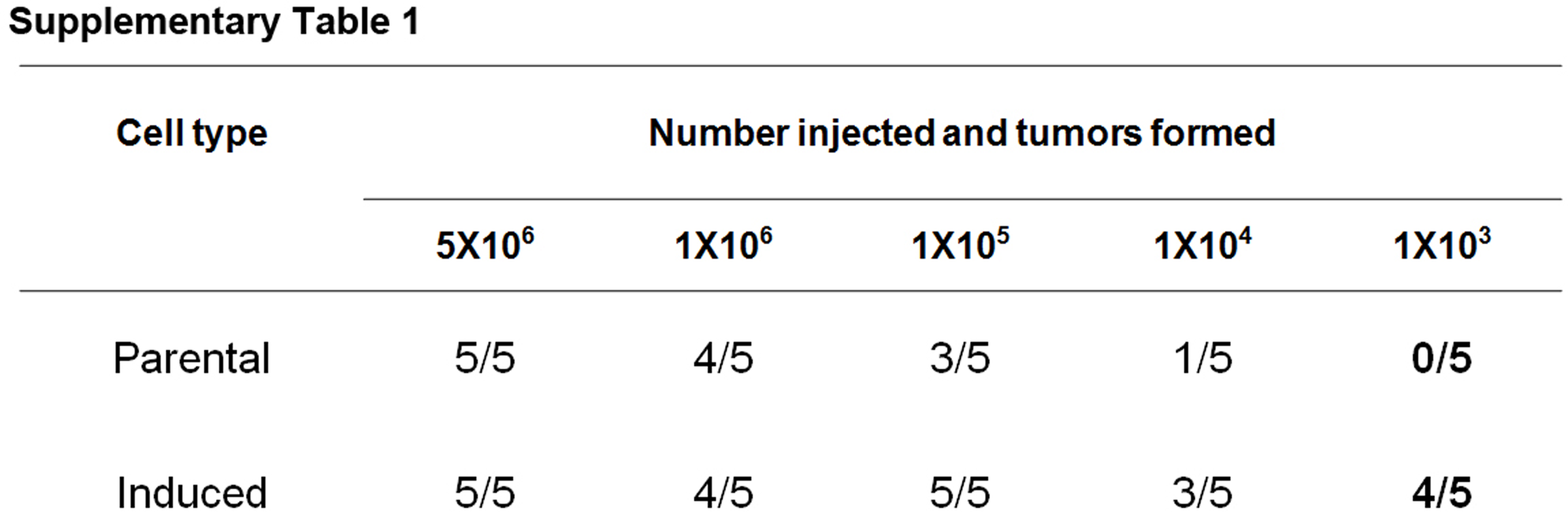

Supplement: Supplementary Table S1 [file cddis2015171x9.tif]
